# Dissecting sources of variability in patient response to targeted therapy: anti-HER2 therapies as a case study

**DOI:** 10.1016/j.ejps.2023.106467

**Published:** 2023-05-16

**Authors:** Timothy Qi, Yanguang Cao

**Affiliations:** a Division of Pharmacotherapy and Experimental Therapeutics, Eshelman School of Pharmacy, The University of North Carolina at Chapel Hill, Chapel Hill, NC 27599, USA; b Lineberger Comprehensive Cancer Center, The University of North Carolina at Chapel Hill, Chapel Hill, NC 27599, USA

**Keywords:** HER2, Tyrosine kinase inhibitor, Breast cancer, Free drug hypothesis, Population pharmacokinetics, Tumor evolution

## Abstract

**Background and purpose:**

Despite their use to treat cancers with specific genetic aberrations, targeted therapies elicit heterogeneous responses. Sources of variability are critical to targeted therapy drug development, yet there exists no method to discern their relative contribution to response heterogeneity.

**Experimental approach:**

We use HER2-amplified breast cancer and two agents, neratinib and lapatinib, to develop a platform for dissecting sources of variability in patient response. The platform comprises four components: pharmacokinetics, tumor burden and growth kinetics, clonal composition, and sensitivity to treatment. Pharmacokinetics are simulated using population models to capture variable systemic exposure. Tumor burden and growth kinetics are derived from clinical data comprising over 800,000 women. The fraction of sensitive and resistant tumor cells is informed by HER2 immunohistochemistry. Growth rate-corrected drug potency is used to predict response. We integrate these factors and simulate clinical outcomes for virtual patients. The relative contributions of these factors to response heterogeneity arecompared.

**Key results:**

The platform was verified with clinical data, including response rate and progression-free survival (PFS). For both neratinib and lapatinib, the growth rate of resistant clones influenced PFS to a higher degree than systemic drug exposure. Variability in exposure at labeled doses did not significantly influence response. Sensitivity to drug strongly influenced responses to neratinib. Variability in patient HER2 immunohistochemistry scores influenced responses to lapatinib. Exploratory twice daily dosing improved PFS for neratinib but not lapatinib.

**Conclusion and implications:**

The platform can dissect sources of variability in response to target therapy, which may facilitate decision-making during drug development.

## 1. Introduction

Patient response to targeted therapy is highly variable and difficult to predict. Multiple factors contribute to diverse patient responses to targeted therapy, including but not limited to drug pharmacokinetics (PK) and biodistribution (Chakrabarti and Michor, 2017), tumor growth characteristics (Weedon-Fekjær et al., 2008), clonal composition (Zhang et al., 2017), and intrinsic sensitivity or resistance to drug (Hafner et al., 2016). Quantitatively characterizing sources of response variability can be valuable for decision-making around dose selection, patient stratification, and therapeutic benefit evaluation. However, some of these factors are challenging to characterize during clinical studies. For example, it is implausible to measure tumor growth rates without treatment in a metastatic disease setting. As a result, the relative contribution of these factors to discrepancies in drug efficacy within diverse patient populations remains largely undefined. Simulations informed by high volumes of historical clinical data and drug potency parameters measured during early-stage drug discovery can be convenient and intuitive tools for dissecting sources of variability in patient response and informing decision-making during drug development.

Here, we propose a pharmacokinetics/pharmacodynamics/tumor growth (PK/PD/TG)-based framework for quantitatively deconvoluting sources of response variability that can be implemented prior to the initiation of large-scale, registration-directed trials. We use HER2 tyrosine kinase inhibitors (TKI) and HER2-amplified metastatic breast cancer to demonstrate that our approach can help deconvolute whether variability in response depends on tumor-intrinsic features, like growth rate and HER2+ fraction, or drug-specific properties, like PK and biodistribution.

## 1. Methods

### 2.1. Modeling framework overview

**Fig. 1.**
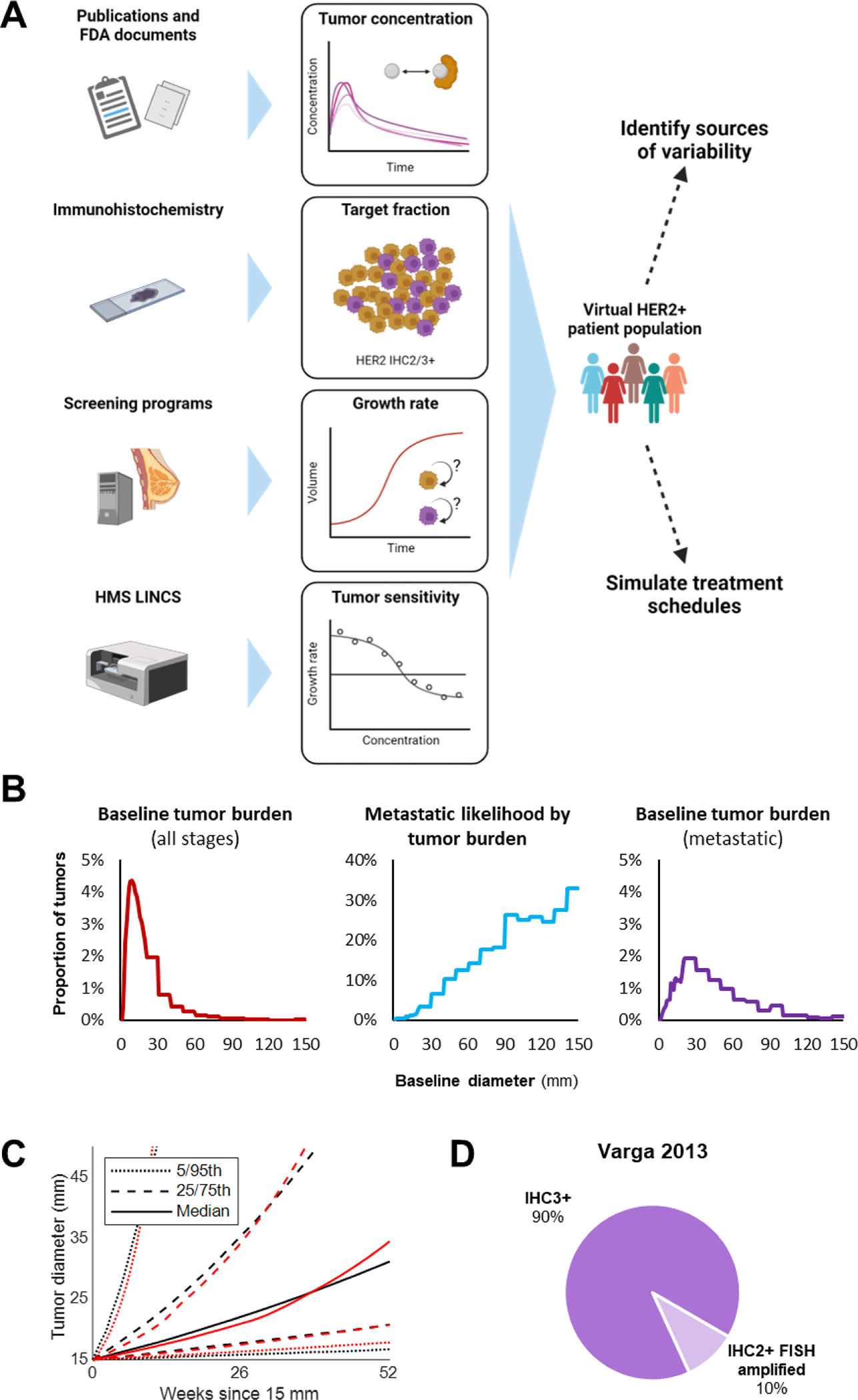
Virtual patients reflect real-world sources of variability A. Variability in drug pharmacokinetics, target fraction, subpopulation growth rates, and drug-specific subpopulation sensitivity were considered during model development. Lapatinib and neratinib population PK models were adapted from publications by the original developers and FDA review documents. Target fraction (subpopulation proportions) was determined using HER2 IHC2/IHC3+ frequencies available in the literature (Varga et al., 2013). Growth rates were derived from a published tumor growth model built on breast cancer screening data from 395,188 women (Weedon-Fekjær et al., 2008). Drug sensitivity for tumor subpopulations was obtained from GR metrics reported in the literature (Hafner et al., 2016), (Mills et al., 2022). B. Baseline tumor diameter at diagnosis (left) and the likelihood of metastatic disease at diagnosis based on baseline tumor diameter (center) used to calculate baseline tumor diameter at diagnosis of metastatic disease (right). Baseline tumor diameter frequencies below 20 mm were available in increments of 1 mm; above 20 mm were available in increments of 10 mm (Sopik and Narod, 2018). C. Simulated growth (red) of 15 mm diameter tumors as compared to (Weedon-Fekjær et al., 2008) (black). Dashed lines indicate 5^th^, 25^th^, 75^th^, and 95^th^ percentiles of 100 simulated tumors, while solid lines indicate medians. D. Relative frequency of IHC3+ and FISH-amplified IHC2+ breast cancers at diagnosis reported in (Varga et al., 2013).

The framework is shown in Fig. 1A. First, we used population PK models to derive drug exposure in patients. Next, we constructed a virtual HER2-positive patient population from large clinical datasets of HER2 IHC and tumor growth kinetics. We then used the in vitro growth rate (GR) metrics to determine sensitivity and resistance of tumor cellular subpopulations to treatment. Finally, we integrated these sources of variability to model the response of tumors with mixed populations to HER2 TKI treatment.

### 2.2. Variability in systemic drug exposure

For neratinib, a population PK model was used to simulate plasma concentration vs. time profiles (Nerlynx (neratinib maleate) Tablets Drug Approval Package 2017). The 2-compartment model included first-order absorption, first-order elimination, and a lag time for absorption. Among all significant covariates, we included the effects of normally distributed age on k_a_ and central volume, as well as the effects of lognormally distributed weight on clearance and central volume. No transporter-mediated uptake into cells was reported. We also assumed that patients would take the drug with a low-fat breakfast under real-world conditions.

For lapatinib, a structurally similar population PK model was used, the only difference being that drug absorption was modeled as a *T*_lag_-delayed zero-order input followed by a first-order input at the rate k_a_ (Zhang and Koch, 2011). We included the effects of normally distributed age on k_a_ and excluded the effects of ethnicity and race on other parameters. No transporter-mediated uptake into cells was reported. We also assumed that patients would take the drug with a low-fat breakfast under real-world conditions.

Free drug concentrations were simulated to inform tumor concentrations based on the Free Drug Hypothesis (Summerfield et al., 2022). Prior to PK/PD simulation, we used the following equation to estimate the average unbound steady-state concentrations of alpelisib (inhibitor of PI3K*α*), abemaciclib (CDK4/6), palbociclib (CDK4/6), ribociclib (CDK4/6), lapatinib (HER1/2), and neratinib (HER1/2/4):

$$C_{ss,free\;}=\frac{X/\tau }{CL/F}\cdot (1-PPB)$$

**Fig. 2.**
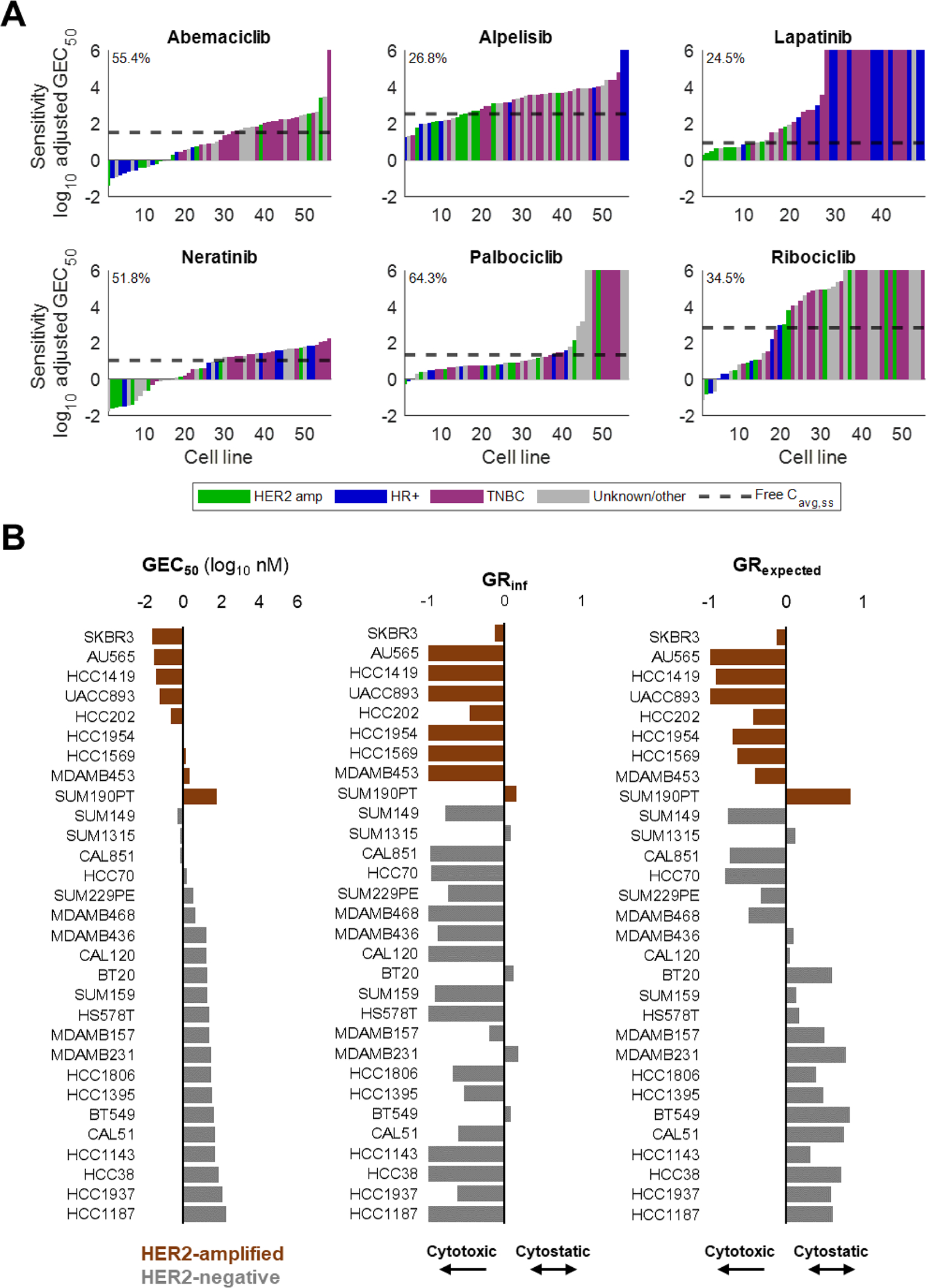
Tumor sensitivity and resistance to targeted therapies is heterogenous A. Protein binding (PPB)-adjusted sensitivity (log_10_ nM) of breast cancer cell lines to six drugs approved for the treatment of metastatic breast cancer (Hafner et al., 2016), (Mills et al., 2022). Dashed lines indicate predicted average steady-state concentrations of free drug (C_ss,free_). Inset percentages indicate the proportion of cell lines in the dataset with PPB-adjusted GEC_50_ below the drug’s predicted free C_ss,free_ (Methods). For visualization purposes, cell lines with no detected sensitivity to a particular drug at tested concentrations were assigned a nominal value of 1 × 10^9^. B. Neratinib GEC_50_ (left) and GR_inf_ (center) values for breast cancer cell lines reported in (Mills et al., 2022). HER2 status was determined (Dai et al., 2017). Cell lines with conflicting HER2 status in the literature were excluded. GR_expected_ (right) values reflect the predicted GR of each cell line at the predicted C_ss,avg_ of neratinib.

Where $C_{ss,\text{free}\;}$ is the steady-state concentration of unbound drug, X is the approved dose, τ is the approved dosing interval, and CL/F is the apparent clearance upon oral administration. PPB is the protein-bound fraction reported in publicly available US Food & Drug Administration (FDA) multidisciplinary review documents for each drug; the exception was neratinib, where the PPB in plasma samples from healthy volunteers was reported to be significantly lower than the in vitro PPB. For lapatinib, the reported PPB of “_>_ 99%” was estimated as 99.5% [(Nerlynx (neratinib maleate) Tablets Drug Approval Package 2017, Tykerb (Lapatinib) Tablets Drug Approval Package 2007)]. The following values were used for each drug: abemaciclib (X=200mg,τ=12hours,CL/F=38L/hour,PPB=96.3%), alpelisib (X=300mg,τ=24hours,CL/F=9L/hour,PPB=89.2%), lapatinib (X=1250mg,τ=24hours,CL/F=114L/hour,PPB=99.5%), neratinib (X=240mg,τ=24 hours, CL/F=195L/hour, in vitro PPB=99%, in vivo PPB=88%), palbociclib (X=125mg,τ=24hours,CL/F=26L/hour,PPB=85%), and ribociclib (X=600mg,τ=24hours,CL/F=26L/hour,PPB=70%). As it is generally believed that only free drug is available to interact with pharmacologically relevant targets in vivo (Summerfield et al., 2022, Tykerb (Lapatinib) Tablets Drug Approval Package 2007), GEC_50_ values in Fig. 2b and A1 were corrected for PPB to estimate sensitivity to free drug concentrations.

### 2.3. Variability in tumor size and composition

To estimate the baseline tumor burden at diagnosis of metastatic disease, we used data from a prior analysis of 819,647 women in the Surveillance, Epidemiology and End Results (SEER) registry (Sopik and Narod, 2018). These data comprise pathologically-confirmed new diagnoses of invasive breast cancer between 1990 – 2014 stored in the SEER 18 registries research database. To calculate the distribution of baseline tumor burden among patients diagnosed with metastatic disease, we multiplied the baseline tumor burden of all patients at diagnosis by the probability of a patient to have metastatic disease given a particular tumor burden (Fig. 1B). Both figures are reported in (Sopik and Narod, 2018). For tumor size probabilities reported over size intervals, we assumed a uniform distribution over the interval and calculated probabilities in diameter increments of 1 mm. A lognormal fit to these data yielded a mean log tumor diameter (mm) of 3.624 and standard deviation of 0.668. Baseline diameters were converted to volumes for simulation, assuming approximately spherical tumors (Fig. 1C). Parameters used for generating virtual populations and treatment simulations are provided in Table A1.

As HER2 TKIs are indicated for patients with HER2+ breast cancer, we needed to estimate the cellular fraction of each tumor that was HER2 amplified. HER2 positivity is currently defined as IHC scores of IHC3+ or IHC2+ with amplification on orthogonal fluorescence in situ hybridization (FISH) assay (Ahn et al., 2020). The proportion of IHC3+ and IHC2+ patients among all HER2+ patients was calculated from 1, 522 samples reported in a 12-year, single-center study (Varga et al., 2013) (Fig. 1D). For simplicity, we assumed each tumor harbored one sensitive (HER2 IHC3+ or IHC2+ with FISH amplification) and one resistant (HER2-negative) population of cells with different levels of sensitivity to HER2 TKIs. Of note, these samples were scored according to older guidelines wherein IHC2+ constitutes HER2+ staining in >_=_ 10% and IHC3+ constitutes HER2+ staining in >_=_30% of tumor cells. Modern-day guidelines indicate that staining in >_=_10% of cells should be considered IHC3+ (Ahn et al., 2020).

### 2.4. Variability in tumor sensitivity and resistance

Once a virtual patient was designated as IHC3+ or IHC2+, a fraction $f_{sens}$ of their tumor cells was designated as sensitive, while $f_{res}=1-f_{sens}$ was designated as resistant. By definition, 10 – 30% of cancer cells in IHC2+ tumors are HER2 amplified, compared to 30 – 100% of cells within IHC3+ tumors (Ahn et al., 2020). Within these two strata, we assumed HER2 amplification was uniformly distributed.

To calibrate the effect of drug concentrations on tumor growth, the in vitro growth rate (GR) metrics of 75 human breast cancer cell lines under treatment with alpelisib, abemaciclib, palbociclib, ribociclib, lapatinib, and neratinib were identified from the literature (Hafner et al., 2016, Mills et al., 2022). Data are presented in Fig. 2A. For use in the model, the HER2 status and clinical subtype of each cell line was cross-referenced with data from (Dai et al., 2017). Cell lines with unknown or inconsistent HER2+ status across publications were excluded. Drug potency (GEC_50_), efficacy (GR_inf_), and Hill coefficient parameters of HER2+ cell lines were sampled to generate HER2 TKI-sensitive cell populations, while the parameters for Basal A and Basal B cell lines were sampled to generate resistant populations. GEC_50_ values were adjusted for PPB as described above. The GR metrics for neratinib and lapatinib are presented in Fig. 2B and A1, respectively.

### 2.5. Modeling responses to HER2 TKI

Tumor growth with and without treatment was modeled using a generalized logistic model (Fig. 1C) (Spratt et al., 1996), which has been validated to faithfully characterize the growth kinetics of breast tumors in a study of 395,188 women (Weedon-Fekjær et al., 2008). These women were aged between 50 – 69, participants of the Norwegian Breast Cancer Screening Program, and had new diagnoses of primary breast cancer. The *b* growth parameter estimated in this study was lognormally distributed with a mean of 1.38 and variance of 1.36 for women under the age of 60 (Weedon-Fekjær et al., 2008). This corresponds to a geometric mean growth rate *k*_*g*_ = 1.66 × 10^−4^ hours, or 2.78 × 10^−2^ weeks. Using this value for *k*_*g*_, the equations used to model changes the growth of sensitive and resistant cell populations over time were:

$$\frac{dN_{sens}}{dt}=k_{g,sens}N_{sens}{\left(1-\frac{N_{sens}\;+\;N_{res}}{K}\right)}^{4}log_{2}\left(1+\;Effect_{sens}\right)$$

$$\frac{dN_{res}}{dt}=k_{g,res}N_{res}{\left(1-\frac{N_{sens}\;+\;N_{res}}{K}\right)}^{4}log_{2}\left(1+\;Effect_{res}\right)$$

$$\frac{dTumor}{dt}=\frac{dN_{res}}{dt}+\frac{dN_{sens}}{dt}$$

Where $k_{g}$ is the growth rate of each population, $N_{\mathrm{sens}}$ and $N_{\mathrm{res}}$ represent the abundance of the sensitive and resistant populations, and K represents the carrying capacity. These equations describe two populations growing toward a shared carrying capacity while impeded by drug. The exponent controls the population density at which crowding begins to significantly slow growth and was previously estimated to be 4 (Weedon-Fekjær et al., 2008, Spratt et al., 1996). The carrying capacity was assumed to equal the largest tumor observed among 395,188 women, which had a diameter of 128 mm (Weedon-Fekjær et al., 2008). Effect_sens_ and Effect_res_ represent the effects of drug on tumor growth and were calculated as follows (Hafner et al., 2016):

$$Effect=GR_{inf}+\frac{1\;-\;GR_{inf}}{1\;+\;{\left(\frac{Free\;drug}{GEC_{50}}\right)}^{h}}$$

Where ${GR}_{\text{inf}}$ and ${GEC}_{50}$ are the growth rate-corrected sensitivities to drug described in Section 1.2.4, Free drug is the unbound drug concentration, and h is a Hill coefficient estimated for a given cell line’s relationship between Free drug, ${GR}_{\text{inf}}$, and ${GEC}_{50}$. This is a generic dose-response relationship derived by (Hafner et al., 2016). Of note, a proportion (27%) of neratinib’s therapeutic effect is attributed to active metabolite exposure (Nerlynx (neratinib maleate) Tablets Drug Approval Package 2017). We therefore multiplied free neratinib concentrations by 1 / (1 – 0.27) when calculating Effect_sens_ and Effect_res_ to adjust for expected metabolite activity.

### 2.6. Analysis and software

Virtual populations were generated in MATLAB R2020b. Treatment simulations were performed in Simulx 2020. Statistical analyses were performed in MATLAB R2020b. Diameters were “measured” by sampling from simulations every 6 or 8 weeks to assess for progression-free survival (PFS), mirroring the frequency of radiographic assessment in clinical trials. PFS was evaluated per RECIST v1.1 criteria, which designate target lesion diameter increases of ≥ 20% from nadir and at least 5 mm in absolute terms as progressive disease (Eisenhauer et al., 2009). Importantly, events such as the appearance of new metastases, enlargement of non-target lesions, and death from any cause were also classified as progressive disease. We termed these events “DNTP”, Death or Non-target Progression, and modeled the daily probability of patients to progress due to DNTP at the empirically estimated rate of 1.5 × 10^−4^ events/mm of tumor diameter/day.

Objective response rates (ORR) were evaluated per RECIST v1.1. This required the calculation of best overall response (BOR) for each patient. To do so, diameter measurements sampled from simulations every 6 or 8 weeks were compared to baseline tumor diameters. The smallest non-baseline tumor measurement was used to evaluate BOR. Patients with BOR diameter reductions of at least 30% from baseline were considered responders. ORR was calculated by dividing the fraction of responders by the total number of patients.

## 3. Results

### 3.1. Tumor sensitivity and resistance to targeted therapies is heterogeneous

We initially questioned whether the steady-state unbound concentrations of several targeted therapies at their approved doses were above or below their in vitro potency against breast cancer cell lines, as measured by PPB-adjusted GEC_50_. Plotting the PPB-adjusted GEC_50_ of breast cancer cell lines (Hafner et al., 2016, Mills et al., 2022) against the estimated C_ss,free_ (Fig. 2A, see Methods) of 6 different drugs revealed significant differences in predicted response. The majority of cell lines in the dataset had GEC_50_ values below the C_ss,free_ of abemaciclib, neratinib, and palbociclib, but not alpelisib, lapatinib, and ribociclib (Fig. 1). While these cell lines may not fully reflect the mixed cellular populations in patients, it is reassuring that HER2-amplified cell types in particular tended to have sensitivities below the predicted C_ss,free_ of HER2 TKIs neratinib and lapatinib. No such enrichment of HER2-amplified cell lines was noted for the CDK4/6 inhibitors abemaciclib, palbociclib, and ribociclib, nor for the PI3Kα inhibitor alpelisib. These data support the fidelity of GR metrics for characterizing and discriminating sensitivity and resistance to HER2 TKIs across diverse cell lines.

### 3.2. Free neratinib in plasma drives patient response and confers therapeutic effect beyond target lesions

**Fig. 3.**
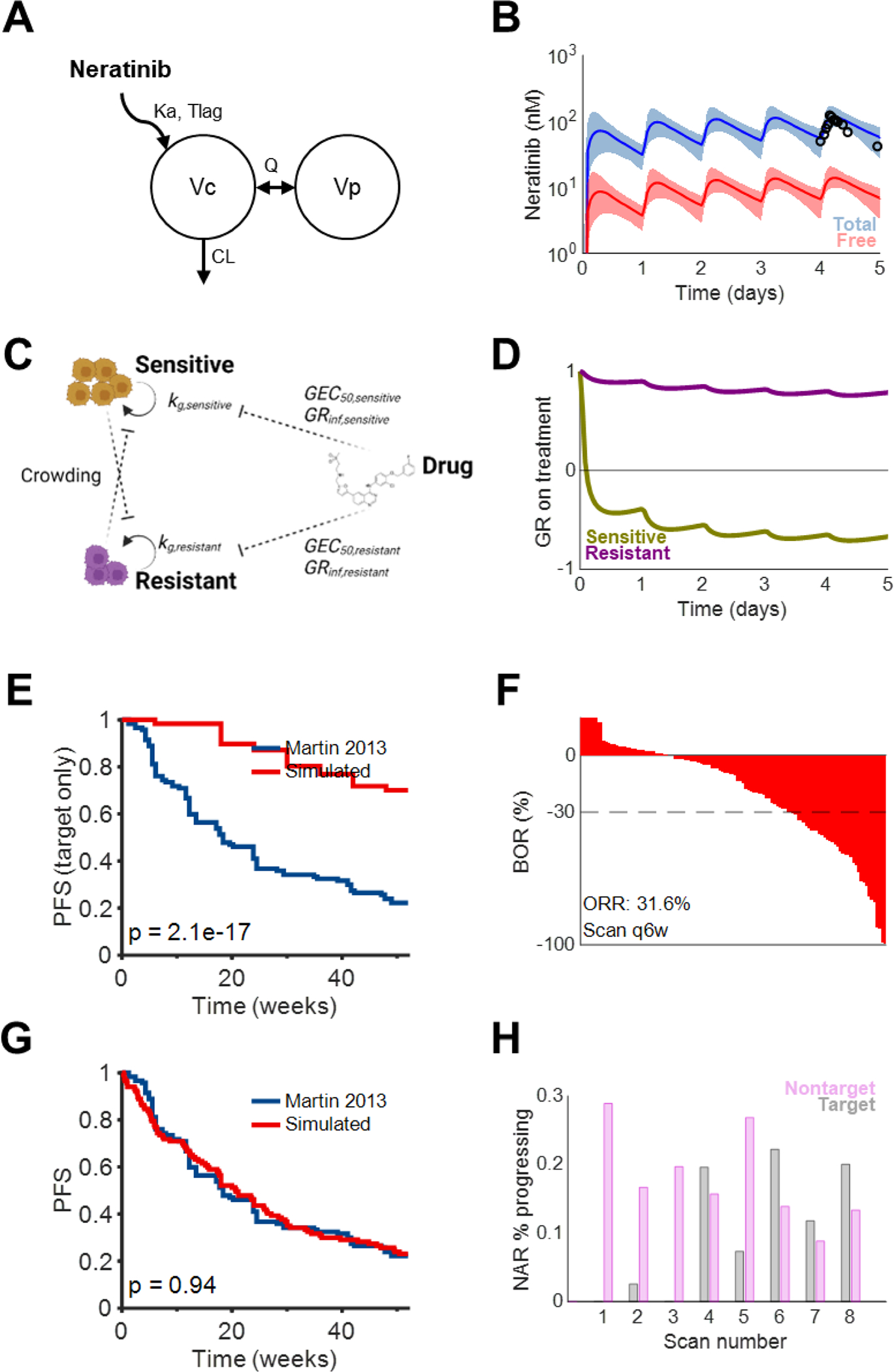
PK/PD/TG model recapitulates the clinical efficacy of neratinib A. Population pharmacokinetic model structure for neratinib reported in (Nerlynx (neratinib maleate) Tablets Drug Approval Package 2017). Drug absorption occurs through a first-order (K_a_) process with delay (T_lag_). Drug subsequently distributes (Q) between central/plasma (V_c_) and peripheral (V_p_) compartments and clears from the central compartment by first-order elimination (CL). B. Simulated pharmacokinetic profiles during the first week of once-daily treatment with 240 mg neratinib. Lines represent medians, whereas shaded regions represent 5^th^ and 95^th^ percentiles. Black circles represent observations from (Keyvanjah et al., 2019). C. Pharmacodynamic model of tumor growth comprising two competing cell populations with differential sensitivity to a cytostatic drug. D. Growth dynamics of HER2-amplified (“sensitive”) and HER2-negative (“resistant”) subpopulations within an illustrative IHC2+ tumor. Values for either subpopulation are normalized to their abundance at treatment initiation. E. Predicted PFS during 52 weeks of treatment with 240 mg QD neratinib using free drug-driven pharmacodynamics, compared to (Martin et al., 2013). Only progression from target lesions is included. F. Predicted ORR during 5 weeks of treatment with 240 mg QD neratinib using free drug-driven pharmacodynamics, compared to (Martin et al., 2013). G. (E) with an additional daily chance of Death or Non-target Progression (DNTP) estimated as 1.5 × 10^−4^ events/mm of tumor diameter/day. H. Risk of progression by source during each 6-week scan interval during 52 weeks of treatment with 240 mg QD neratinib. NAR, number at risk during the scan interval.

We created a virtual HER2+ breast cancer population and simulated patient response to neratinib (see Methods). The population PK of neratinib was simulated using a two-compartment model with sequential first-order absorption, T_lag_, and first-order elimination (Fig. 3A–B) [(Nerlynx (neratinib maleate) Tablets Drug Approval Package 2017, Martin et al., 2013, Keyvanjah et al., 2019)]. A generalized logistic growth model was used to describe the growth of a two-population tumor with different subpopulation sensitivities to neratinib (Fig. 3C–D) (Weedon-Fekjær et al., 2008, Spratt et al., 1996).

We explored several possibilities for tumor suppressive effects, including total- and free-drug concentration-driven tumor growth inhibition of target lesions as well as indirect (unobserved) suppression of the growth of non-target and new lesions. When only target lesions were considered, tumor growth inhibition driven by total drug concentrations resulted in an overestimation of PFS and ORR (Fig. A2) (Martin et al., 2013). This was not entirely unexpected, as progression from target lesions only reflects a subset of patients; many breast cancer progression events are due to death, new metastases, or non-target lesion progression (Litière et al., 2014). Using free neratinib concentrations improved concordance and matched the target lesion response rates reported in (Martin et al., 2013) (31.6% simulated vs. 30.3% reported), but still overestimated PFS (Fig. 3E–F).

We found the inclusion and drug-mediated suppression of non-target and new lesions to be critical for producing clinically consistent outcomes. For tumors that are large at baseline, modeling drug effect on target lesions alone may overpredict patient response, as these patients are at higher risk of non-target progression and new lesion appearance. Considering (1) the positive correlation between tumor burden and mortality and/or metastasis (Sopik and Narod, 2018), and (2) that more patients with metastatic breast cancer progress from non-target sources than target sources (Litière et al., 2014), we derived an additional time-variant probability of Death or Non-target progression (DNTP) for each patient that increased as a function of tumor burden. DNTP represents the instantaneous daily probability of a patient to die, experience new metastasis, or exhibit non-target lesion growth and was estimated as 1.5 × 10^−4^ events/mm tumor diameter/day.

With free drug-driven tumor growth inhibition and tumor burden-driven DNTP, PFS estimates matched well with clinical outcomes (Martin et al., 2013) (Fig. 3G). Interestingly, the risk of progression from non-target, DNTP sources was relatively consistent throughout the course of treatment, whereas the risk of progression from target lesions was highest toward the end of treatment (Fig. 3H). Considering these results, free drug-driven PD with concomitant DNTP was carried forward for all subsequent simulations.

### 3.3. Response to neratinib is mostly influenced by tumor characteristics and less by systemic drug exposure

**Fig. 4.**
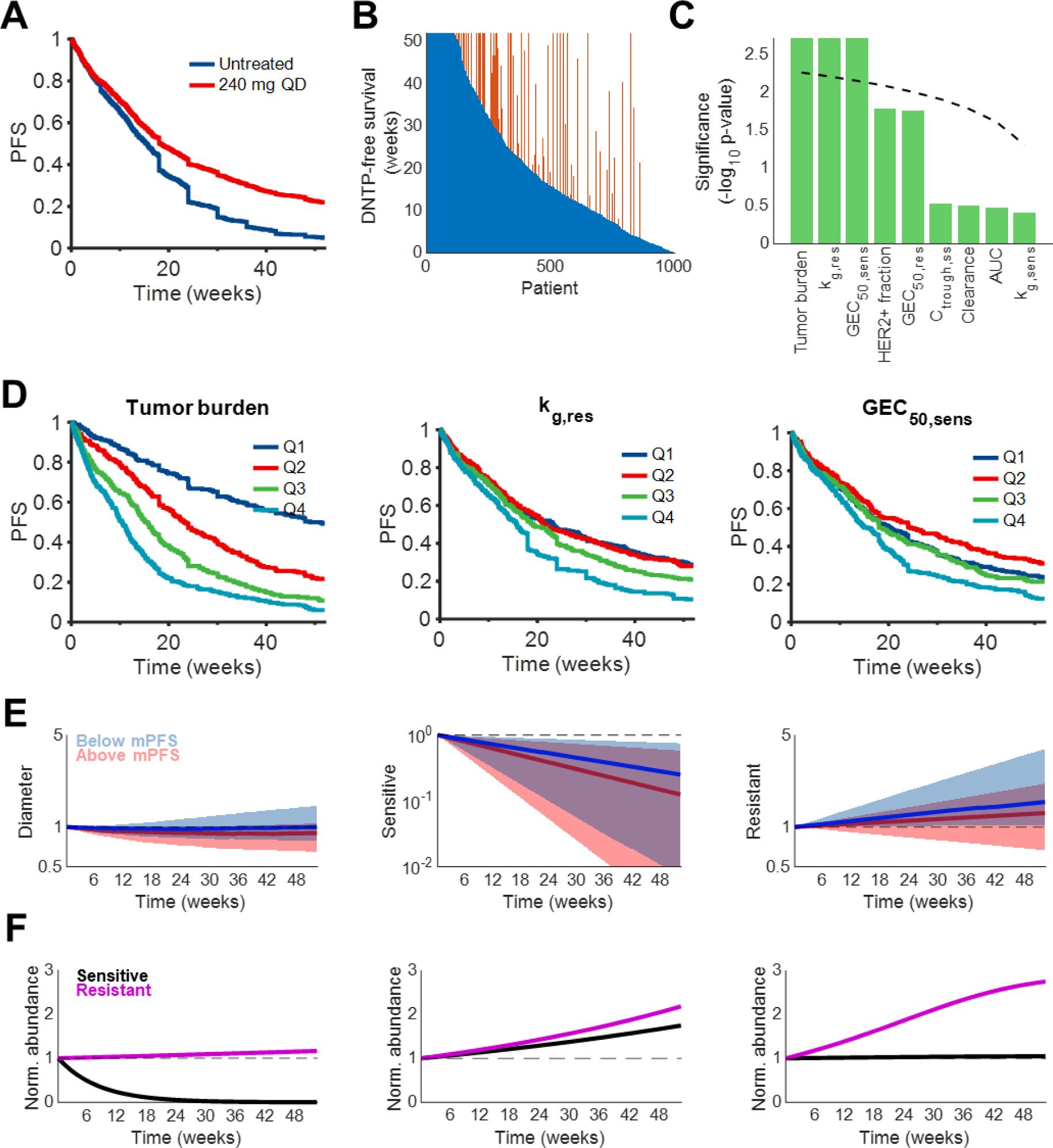
Tumor-intrinsic characteristics influence neratinib efficacy A. Simulated PFS of 1000 patients with and without 52 weeks of treatment with 240 mg QD neratinib. B. DNTP-free survival for each patient in untreated (blue) and treated (orange) states. DNTP-free survival is the time on treatment without a DNTP event. Each orange bar represents a patient whose DNTP-free survival was extended by therapy. C. Multiplicity-corrected significance of differences in PFS between patients with parameter values above or below the population median (bars). The dashed line represents the Benjamin-Hochberg significance threshold for one-way ANOVA. For visualization purposes, the y axis is truncated at 2.5. D. Significant differences in PFS stratified by tumor burden (left), resistant cell growth rate (middle), and sensitive cell GEC_50_ (right) quartiles. E. Normalized tumor diameters (left), sensitive cell volume (middle), and resistant cell volume (right) stratified by length of PFS relative to median PFS (mPFS). All y axes are on a log scale. Shaded regions represent interquartile ranges. F. Representative profiles of baseline-normalized sensitive and resistant cell abundance from three virtual patients treated with 52 weeks of 240 mg QD neratinib.

Having established the model’s ability to reasonably recapitulate the clinical outcomes of neratinib, we proceeded to simulate 52 weeks of neratinib monotherapy in 1,000 virtual patients in search of tumor characteristics associated with longer PFS. Treatment with 240 mg QD neratinib prolonged median PFS from 15.5 to 18.3 weeks (Fig. 4A) and extended DNTP-free survival in 25.7% of patients (Fig. 4B).

Patients were stratified by tumor characteristics and systemic drug exposure (Fig. 4C). As expected, greater baseline tumor burden correlated with shorter PFS (median PFS in Q1, 49 weeks; Q2, 23.1 weeks; Q3, 15.1 weeks; Q4, 10.4 weeks) (Fig. 4D). The growth rate of resistant cells (median PFS in Q1, 23.1 weeks; Q2, 21.3 weeks; Q3, 19.1 weeks; Q4, 15.7 weeks) and drug potency on sensitive cells (median PFS in Q1, 20.1 weeks; Q2, 24.6 weeks; Q3, 18 weeks; Q4, 15 weeks) were also great sources of variability. Patients with more sensitive cell populations or slower growth of resistant populations tended to have longer PFS. In contrast, the growth of sensitive cell populations, drug potency against resistant cell populations, and drug PK parameters like clearance, area under the curve (AUC), and C_ss,trough_ at the labeled dose did not significantly influence patient response and PFS (Fig. 4C). Interestingly, differences in HER2+ fraction were also minimally informative of patient response.

Patients who achieved above-median PFS tended to have greater killing of sensitive cells and better control of resistant cell outgrowth than patients who did not (Fig. 4E). On an individual level, however, a diverse range of response dynamics was observed (Fig. 4F). Sensitive subpopulations were completely eradicated in some patients, fully controlled in others, and completely insensitive in still others (Fig. 4F), raising the possibility of post-treatment changes to HER2+ fraction (Bon et al., 2020). Collectively, these data suggest that tumor-intrinsic characteristics and cellular heterogeneity influence patient responses to targeted therapy to a greater degree than inter-individual variability in drug PK at approved doses.

### 3.4. Patient response to lapatinib is also largely explained by variability in tumor characteristics and cellular composition

**Fig. 5.**
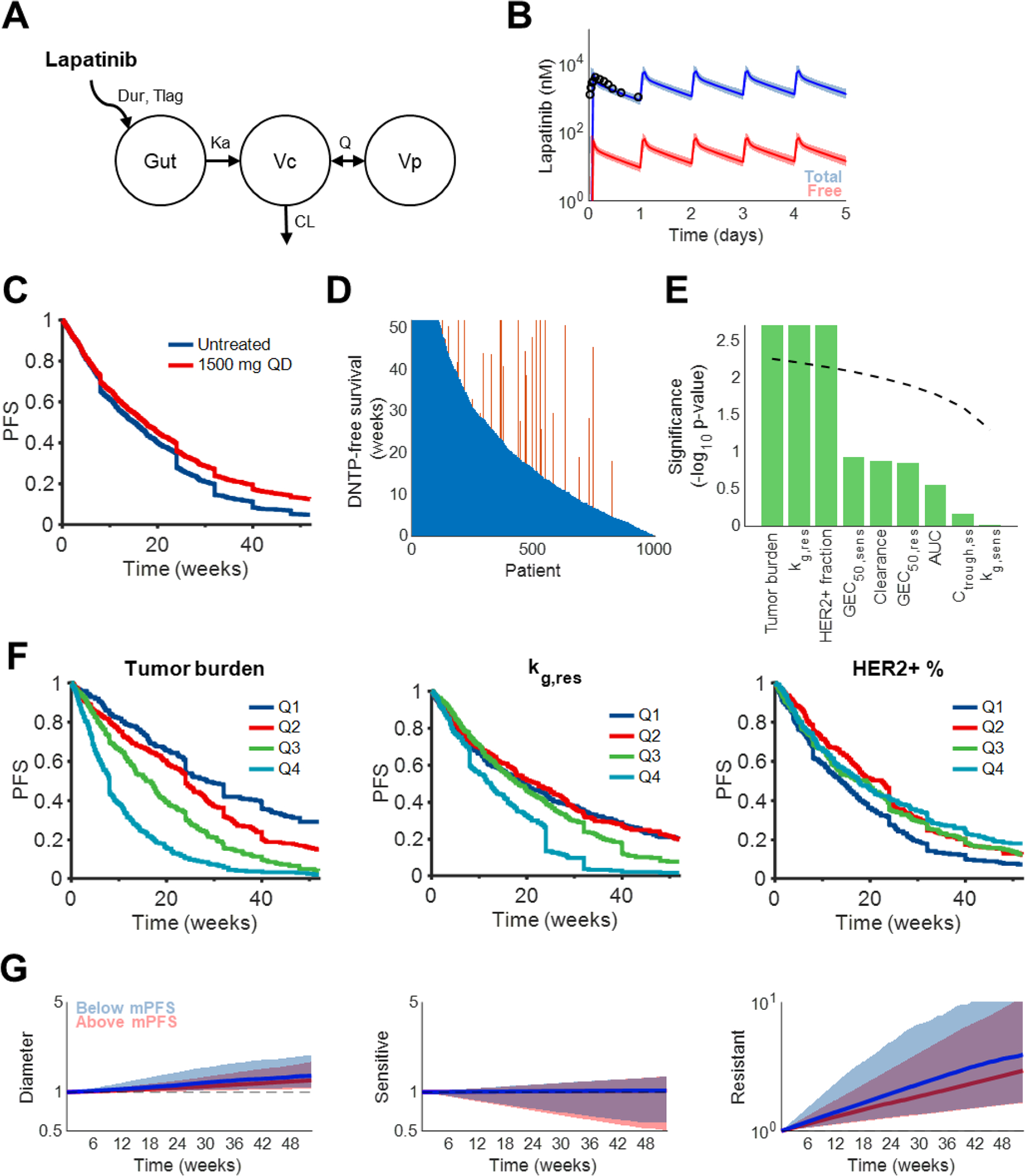
Different tumor-intrinsic characteristics influence lapatinib efficacy A. Population pharmacokinetic model structure for lapatinib reported in (Zhang and Koch, 2011). Drug absorption occurs through a sequential zero-order (Dur) and first-order (K_a_) process with delay (T_lag_). Drug subsequently distributes (Q) between central/plasma (V_c_) and peripheral (V_p_) compartments and clears from the central compartment by first-order elimination (CL). B. Simulated pharmacokinetic profiles during the first week of once-daily treatment with 1500 mg lapatinib. Lines represent medians, whereas shaded regions represent 5^th^ and 95^th^ percentiles. Black circles represent observations from (Koch et al., 2009). C. Simulated PFS of 1000 patients with and without treatment of 1500 mg daily lapatinib. D. DNTP-free survival for each patient in untreated (blue) and treated (orange) states. DNTP-free survival is the time on treatment without a DNTP event. Each orange bar represents a patient whose DNTP-free survival was extended by therapy. E. Multiplicity-corrected significance of differences in PFS between patients with parameter values above or below the population median (bars). The dashed line represents the Benjamin-Hochberg significance threshold for one-way ANOVA. For visualization purposes, the y axis is truncated at 2.5. F. Significant differences in PFS stratified by tumor burden (left), resistant cell growth rate (middle), and sensitive cell HER2+ fraction (right) quartiles. G. Normalized tumor diameters (left), sensitive cell volume (middle), and resistant cell volume (right) stratified by length of PFS relative to median PFS (mPFS). All y axes are on a log scale. Shaded regions represent interquartile ranges.

To investigate the broader applicability of our modeling approach, we adjusted the PK/PD/TG model for another HER2 TKI, lapatinib (Fig. 5A). Simulations using the virtual breast cancer population, publicly available GR metrics (Hafner et al., 2016, Mills et al., 2022), and the original sponsor-developed population PK models reproduced the clinically observed PK, PFS, and ORR of 1500 mg QD lapatinib (Fig. 5B and A3) (Zhang and Koch, 2011, Toi et al., 2009, Burstein et al., 2008, Koch et al., 2009). As with neratinib, free plasma lapatinib was assumed to drive tumor suppression.

Lapatinib monotherapy was predicted to improve median PFS to 17.4 weeks, from 15.1 weeks if untreated (Fig 5C). DNTP-free survival was also extended in 11.4% of patients (Fig. 5D). Similar to neratinib, differences in PFS were observed for key tumor-intrinsic properties such as tumor burden (median PFS in Q1, 28.4 weeks; Q2, 24 weeks; Q3, 15.7 weeks; Q4, 8 weeks) and resistant cell growth rate (median PFS in Q1, 19.4 weeks; Q2, 22.4 weeks; Q3, 18.3 weeks; Q4, 12.4 weeks), while relationships between PFS and PK parameters were absent (Fig. 5E). HER2+ fraction also impacted lapatinib outcomes, with the lowest expression tumors exhibiting markedly shorter PFS (median PFS in Q1, 13.9 weeks; Q2, 22.4 weeks; Q3, 19.1 weeks; Q4, 17.6 weeks) (Fig. 5F). Sensitivity to drug did not influence PFS. In contrast to neratinib, there was no difference in the killing of sensitive cells between patients who achieved above- and below-median PFS, although there was a difference in the control of resistant cell outgrowth (Fig. 5G).

### 3.5. Dose modifications may improve clinical response for neratinib but may not for lapatinib

**Fig. 6.**
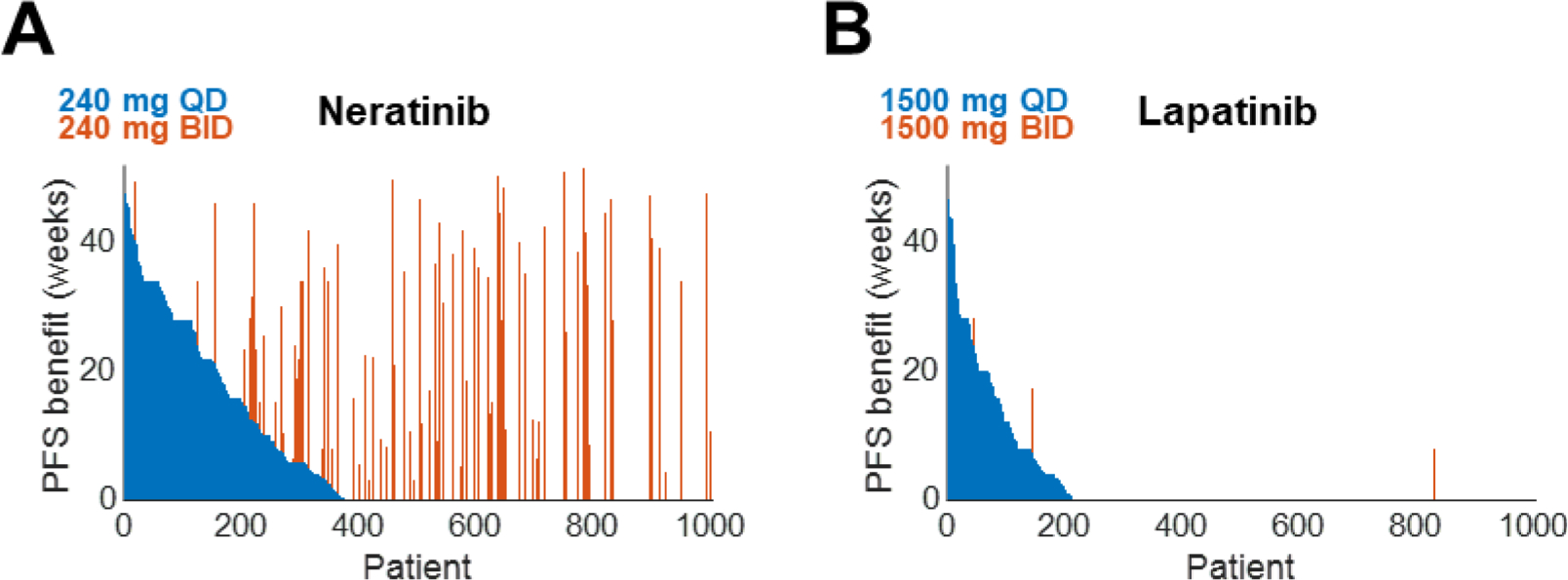
Dose intensification benefits neratinib more than lapatinib A. PFS benefit from treatment with 240 mg QD or BID neratinib versus no treatment. Each orange bar indicates a patient whose PFS was longer on BID dosing compared to QD dosing. B. (A) for 1500 mg QD or BID lapatinib.

Although there was only a modest relationship between PK variability and PFS under the approved doses of neratinib and lapatinib, we wondered whether BID dosing might improve tumor control and patient outcomes. To our surprise, doubling the dosing frequency improved outcomes for neratinib significantly more than for lapatinib, with 27% versus 2% deriving longer PFS from BID dosing (Fig. 6A–B). This suggests different levels of susceptibility of neratinib and lapatinib, at their approved doses, to significant changes in PK; 240 mg QD neratinib is perhaps in a steeper portion of its dose-response curve than 1500 mg QD lapatinib. Of note, simulated dose fractionation of neratinib into 120 mg BID to mitigate dose-limiting diarrhea did not significantly influence efficacy (Fig. A4) (Nerlynx (neratinib maleate) Tablets Drug Approval Package 2017).

## 4. Discussion

In this work, we developed a PK/PD/TG model for decomposing sources of variability between responders and non-responders. We simulated the highly variable PK of two HER2 TKIs, neratinib and lapatinib, and their therapeutic effects on tumor growth and progression. We also modeled a wide range of tumor sensitivity to therapy as informed by drug potency in cell lines with varying HER2 expression and population-level diversity in HER2 immunohistochemistry (IHC) scores. These data were integrated into a virtual patient population to dissect the sources of variability in patient response.

There are two key aspects of our approach. One is the use of population PK models to simulate drug PK and variability in patients. Population PK models and parameter estimates for investigational and approved drugs are usually readily available to sponsors. Using the drug sponsors’ population PK models ensured that our simulations could recapitulate a wide range of possible drug exposures. This is of critical importance, as drug exposure can vary dramatically between patients. Second, we modeled a mixed tumor comprising subpopulations of sensitive (HER2-amplified) and resistant (HER2-negative) cells, with relative abundances informed by patient HER2 IHC scores. Subpopulation-specific cytostatic and cytotoxic effects were applied using the growth rate inhibition metrics developed by Hafner et al. (Hafner et al., 2016). These metrics correct for differences in growth rates among cell lines, which can otherwise confound interpretations of drug potency. Recent publications showcase the added value of using growth rate metrics for predicting in vivo responses from in vitro data alone (Diegmiller et al., 2022) and support the further elaboration of this approach for drug development.

Previous studies conducted to evaluate the interplay between PK and tumor characteristics have found both to be major sources of variability in patient response (Chakrabarti and Michor, 2017). While we also found tumor growth parameters such as resistant cell growth rate to contribute significantly to PFS, we did not find a strong influence of drug PK on patient response to the assessed targeted therapies.

The finding that HER2+ positivity influenced clinical outcomes under lapatinib treatment is consistent with clinical observations (Filho et al., 2021). It is not clear why a similar magnitude of effect was not observed in patients treated with neratinib. We also found the growth rate of resistance clones, analogous to tumor regrowth rate after progression, to be significant to patient response. Interestingly, growth rate after progression has been directly correlated with patient survival in non-small cell lung cancer and glioblastoma (Nishino et al., 2021, Yong et al., 2014). Though a similar analysis has been conducted for HER2-negative breast cancer response to chemotherapy (Krishnan et al., 2021), no such work exists for HER2-positive breast cancer to our knowledge. Our results, in the context of accumulating literature highlighting the importance of HER2+ fraction, suggest a similar relationship between regrowth rate and long-term outcomes may exist.

Including a tumor burden-based risk of DNTP was critical for recapitulating clinically observed PFS curves, which account for progression from both target and non-target sources (Sopik and Narod, 2018, Litière et al., 2014). Patients with metastatic disease may have discordant responses to therapy across metastases, confounding PFS based on changes in the target lesions (Zhou et al., 2021, Zhou et al., 2020). To our knowledge, DNTP rates have not been well characterized previously. Our estimate of DNTP was robust to patient censoring in the clinical benchmark datasets, perhaps due to the relative infrequency of censoring: only 5.4% and 13.7% of patients on lapatinib or neratinib were censored, respectively. Sensitivity analyses indicated that the true rate of DNTP could have ranged between 1.25 – 1.8 × 10^−4^ events/mm tumor diameter/day, which is within 30% of the value we used in our simulation (Fig. 5A). However, it is critical to recognize that this rate likely varies across indications, given fundamental differences in tumor aggressiveness and metastatic lability. Going forward, efforts to model DNTP rates may be informed by the difference in progression between target lesions alone versus all lesions. These efforts should also evaluate the impact of censoring on estimated values and report a range of PFS outcomes.

Two points should be kept in mind when interpreting this study. First, nonspecific protein binding is much higher in tumors than in blood for most small molecule therapeutics. For lapatinib, Spector et al., 2015 showed that total drug concentrations in xenograft tumors are slower clearing and at least 6-fold higher than in plasma. With substantial total drug accumulation in the tumor due to nonspecific binding, one would expect total drug-driven effect to result in even longer PFS than we have simulated here, therefore requiring a higher rate of DNTP to “bring down” the PFS curve. One major assumption of this work is that nonspecific proteins available for drug binding are always in great excess, leading to a constant f_up_ over time (Meijer and Van der Sluijs, 1987, Sparreboom et al., 2001). Hypoalbuminemia does occur in breast cancer, although whether this meaningfully affects free drug concentrations is not known (Fujii et al., 2020).

Second, the Free Drug Hypothesis is generally taken *prima facie* when translating drug PD from in vitro systems into living organisms (Summerfield et al., 2022, Diegmiller et al., 2022). This draws from the assumption that drug nonspecifically bound to circulating plasma protein or extracellular parenchymal protein is unable to interact with its intended target – in this case, the intracellular kinase domain of HER2. Violations of the free drug hypothesis are generally driven by mechanisms that disturb steady state equilibria assumptions, such as drug transport proteins like P-glycoprotein (P-gp) or the presence of high tissue clearance rates relative to diffusion (Summerfield et al., 2022). While lapatinib is a substrate for P-gp and breast cancer resistance protein (BCRP) (Polli et al., 2008), the direction of its transport is extracellular, such that intracellular lapatinib concentrations are unlikely to be higher than what one would expect from free plasma concentrations. With no evidence to suggest transporter-mediated uptake into cancer cells, nor increased clearance from the tumor, we consider free drug concentrations to be an appropriate driver of PD for lapatinib [(Nerlynx (neratinib maleate) Tablets Drug Approval Package 2017, Tykerb (Lapatinib) Tablets Drug Approval Package 2007, Spector et al., 2015)]. Hypothetically, covalent inhibitors such as neratinib might cause receptor internalization, leading to longer PD than would be expected from free concentrations (Li et al., 2020). If de novo HER2 re-synthesis kinetics are faster than neratinib’s half-life, however, the prolongation of PD would be negligible (Zhang et al., 2019).

Oncology drug developers may benefit from carefully considering free drug concentrations when predicting clinical efficacy from pre- and non-clinical data. With our emphasis on free drug concentrations, it might be tempting to use this study to support optimizing protein binding as a goal during early development. However, this would be misguided, as decreasing protein binding does not increase free drug exposure without a concomitant decrease in intrinsic clearance (Liu et al., 2014). For drugs such as lapatinib, correcting for protein binding and comparing to GR metrics like GEC_50_ may have alerted investigators to the relatively minimal effect lapatinib would exert as a monotherapy (Burstein et al., 2008); indeed, lapatinib is only approved for use in combination with capecitabine. Previous studies that estimated brain tumor lapatinib concentrations did not consider nonspecific protein binding and used a simplified representation of the drug’s pharmacokinetic variability (Stein et al., 2018). Interestingly, the 0.61 tumor-to-blood ratio that was used is lower than the tumor accumulation ratio reported by Spector et al., though this may be due to differences in brain and peripheral drug distribution (Spector et al., 2015). In any case, it may be useful to re-evaluate these studies in the context of blood brain barrier-mediated transport of free drug.

Developing a well-calibrated model with realistic characterization of inter-patient PK and tumor growth variability allowed us to explore alternative dosing regimens in a semi-realistic population. We were surprised to find substantial differences in the extent of benefit achieved with BID neratinib and lapatinib. Hypothetically, this suggests dose titration of lapatinib up to 1500 mg BID would be futile in most cases, whereas 240 mg BID neratinib would be reasonably likely to extend PFS. In practice, dose-limiting diarrhea may prevent the administration of more than 240 mg QD neratinib, highlighting that safety and tolerability can impose upper limits to the range of the free drug ER that can be explored (Nerlynx (neratinib maleate) Tablets Drug Approval Package 2017). Splitting the 240 mg dose into two 120 mg doses did not compromise the efficacy of neratinib (Fig. A4), but did lower C_ss,max_ without changing C_ss,avg_. This may be an alternative to the currently on-label dose adjustment strategy, which recommends sequential dose reductions in increments of 40 mg. FDA review of the ER relationship of neratinib suggested response rates would be almost 50% lower if patients received only 80 mg of neratinib QD (Nerlynx (neratinib maleate) Tablets Drug Approval Package 2017). We note that such a large reduction in exposure is not expected to be within the range of patients taking 240 mg neratinib QD, but nonetheless supports 240 mg QD being in a non-flat region of neratinib’s ER curve. ER for lapatinib was not available.

HER2 was an unusually apt target on which to demonstrate modeling proof of concept, as HER2 amplification is routinely quantified in the clinic. In addition, widespread breast cancer screening efforts have generated volumes of data against which it is possible to calibrate models of tumor growth (Weedon-Fekjær et al., 2008). Based on these results, it may be useful to consider HER2+ fraction as a predictor of outcomes on HER2 targeted therapy. This may provide greater granularity than HER2 IHC scores. An immediate expansion of this approach would be for the prediction of EGFR TKI efficacy, as several groups have found EGFR mutation fraction to influence outcomes during treatment with EGFR mutation-selective therapies (Li et al., 2017, Liu et al., 2022). Overall, our GR metric-based population PK/PD/TG modeling approach has potentially broad utility for decision-making during early drug development.

## Supplementary Material

1 **Figure A1.** Lapatinib drug sensitivity
**Figure A2.** Neratinib pharmacodynamics using total or free drug
**Figure A3.** Lapatinib efficacy calibration
**Figure A4.** Neratinib dose fractionation

2 **Table A1.** Model parameters

3 **Table A2.** Patient characteristics of neratinib monotherapy trials

4 **Table A3.** Patient characteristics of lapatinib monotherapy trials

## Data Availability

The data that support the findings of this study will be made available from the corresponding author upon reasonable request.

## Abbreviations

AUC: area under the curve
BID: twice daily
BOR: best overall response
CDK: cyclin-dependent kinase
DNTP: death or non-target progression
FDA: Food & Drug Administration
FISH: fluorescence in situ hybridization
GEC_50_: concentration at half-maximal growth rate inhibition effect
GR: growth rate inhibition [metrics]
GR_inf_: growth rate inhibition at infinite drug concentration
HER: human epidermal growth factor receptor
HMS LINCS: Harvard Medical School Library of Network-based Cell Signatures
IHC: immunohistochemistry
ORR: objective response rate
PD: pharmacodynamics
PFS: progression-free survival
PI3Kα: phosphoinositide 3-kinase alpha
PK: pharmacokinetics
PPB: plasma protein bound
QD: once daily
TKI: tyrosine kinase inhibitor
