# Supplementary material for: Dissecting sources of variability in patient response to targeted therapy: anti-HER2 therapies as a case study
Source: Eur J Pharm Sci. Author manuscript; Available in PMC 2023 Jul 1. (PMC10290754; doi:10.1016/j.ejps.2023.106467)
Supplement: 1 — Figure A1. Lapatinib drug sensitivity Figure A2. Neratinib pharmacodynamics using total or free drug Figure A3. Lapatinib efficacy calibration Figure A4. Neratinib dose fractionation [file NIHMS1904089-supplement-1.docx]

**Figure A1.** Lapatinib drug sensitivity

1. Lapatinib GEC_50_ (left) and GR_inf_ (center) values for breast cancer cell lines reported in [4]. HER2 status was determined by [13]. Cell lines with conflicting HER2 status in the literature were excluded. GR_expected_ (right) values reflect the predicted GR of each cell line at the estimated C_ss,avg_ of lapatinib.

**
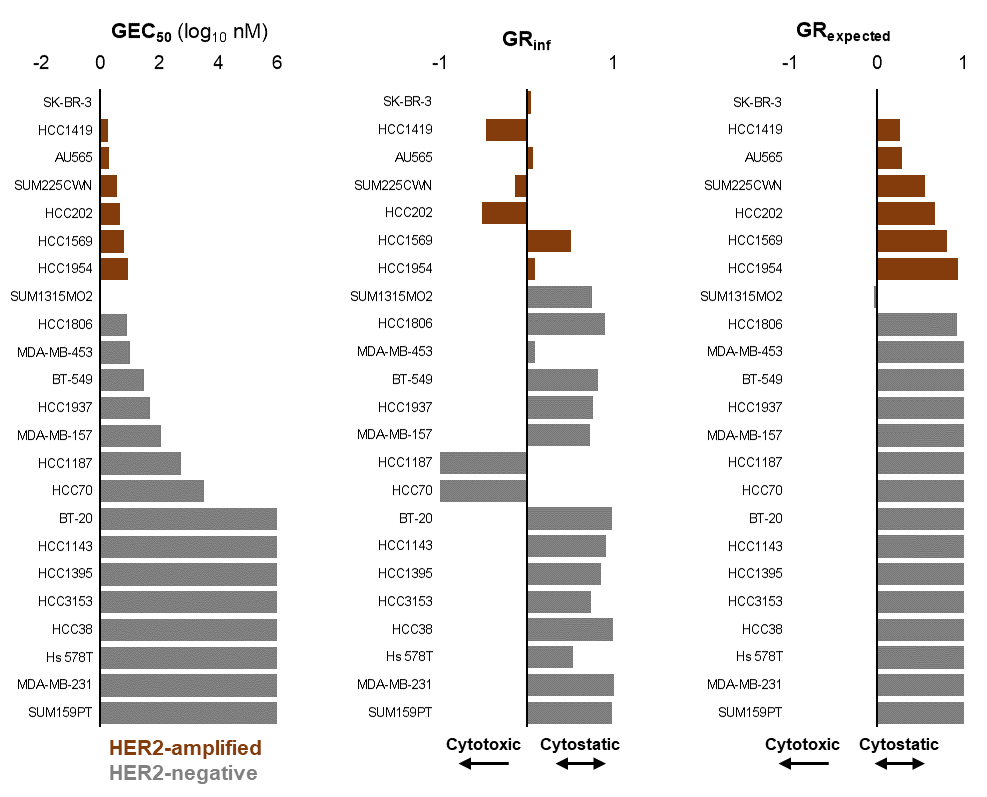
**

**Figure A2.** Neratinib pharmacodynamics using total or free drug

1. Predicted PFS (top) and ORR (bottom) during treatment with 240 mg daily neratinib using total drug-driven pharmacodynamics, compared to [16]. Only progression from target lesions is included.
2. (A) using free drug-driven pharmacodynamics.
3. (B) with an additional daily chance of Death or Non-target Progression (DNTP) estimated as 1.5 x 10^-4^ events/mm of tumor diameter/day.

**
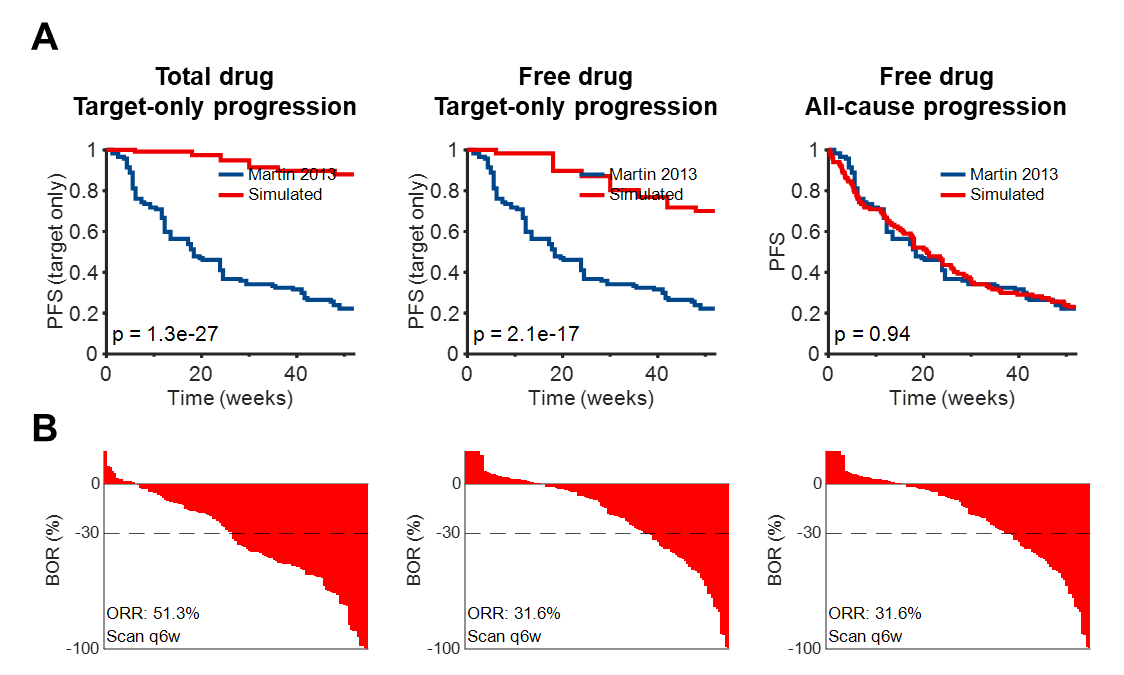
**

**Figure A3.** Lapatinib efficacy calibration

1. Predicted PFS (top) and ORR (bottom) of 1500 mg QD lapatinib using free-driven pharmacodynamics and an additional daily chance of Death or Non-target Progression (DNTP) estimated as 1.5 x 10^-4^ * tumor diameter in mm, compared to [20], [21].

**
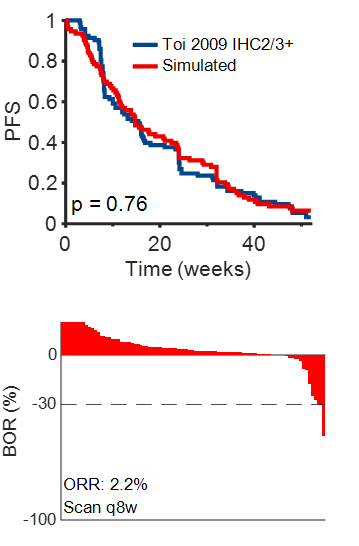
**

**Figure A4.** Neratinib dose fractionation

1. PFS benefit from treatment with 240 mg QD or 120 mg BID neratinib versus no treatment. PFS benefit is the positive difference in predicted PFS between two treatment schedules. Lack of orange bars indicates PFS equivalence between 240 mg QD and 120 mg BID.

**
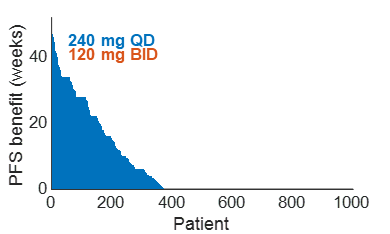
**

**Figure A5.** Robustness to censoring

1. Predicted PFS during 52 weeks of treatment with 1500 mg QD lapatinib (left) 240 mg QD neratinib (right) using free drug-driven pharmacodynamics, with an additional daily chance of Death or Non-target Progression (DNTP) estimated as 1.5 x 10^-4^ events/mm of tumor diameter/day. Clinically observed PFS curves (blue) were adjusted assuming patients progressed at the time of censoring (5, lapatinib; 16, neratinib). Re-estimation of DNTP yielded an optimal value of 1.8 x 10^-4^.
2. (A) with PFS curves adjusted assuming patients censored were event-free for the duration of the trial. Re-estimation of DNTP yielded an optimal value of 1.25 x 10^-4^.

**
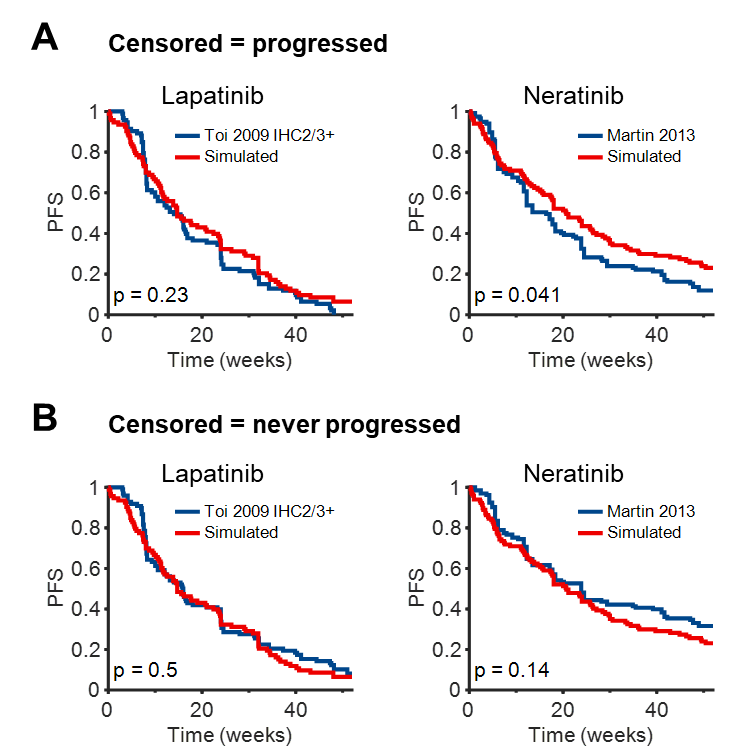
**
